# Characterising Pain in Post-COVID-19 Syndrome: An Observational Study of Intensity and Sensitivity

**DOI:** 10.3390/diagnostics16132023

**Published:** 2026-06-29

**Authors:** Laura Pérez-Gisbert, Gregory Reychler, Beatriz Brea-Gómez, Concepción Morales-García, Marie C. Valenza, Irene Torres-Sánchez

**Affiliations:** 1 Department of Physiotherapy, Faculty of Health Sciences, University of Granada, 18016 Granada, Spain; lauraperezg@ugr.es (L.P.-G.); beatrizbrea@correo.ugr.es (B.B.-G.); cvalenza@ugr.es (M.C.V.); 2 Department of Physiotherapy and Occupational Therapy, Saint-Luc University Clinics, 1200 Brussels, Belgium; gregory.reychler@saintluc.uclouvain.be; 3 Institute of Experimental and Clinical Research (IREC), Pulmonology, ENT and Dermatology Cluster (LuNS, Lung-Nose-Skin), Catholic University of Louvain (UCLouvain), 1200 Brussels, Belgium; 4 Department of Pulmonology, Saint-Luc University Clinics, 1200 Brussels, Belgium; 5 Department of Pulmonology, Virgen de las Nieves University Hospital, 18014 Granada, Spain; concepcion.morales.sspa@juntadeandalucia.es

**Keywords:** COVID-19, post-COVID-19 syndrome, pain intensity, pressure pain threshold, health status

## Abstract

**Background/Objectives**: Post-COVID-19 syndrome (PCS) is frequently accompanied by pain, which may coexist with alterations in multiple health domains. However, pain in PCS has rarely been explored from a multidimensional approach combining subjective and objective measures. Objective: To describe pain in subjects with PCS using pain intensity and the pressure pain threshold (PPT), and to examine the associations between these measures and descriptive characteristics as well as health status. **Methods**: A cross-sectional observational study was conducted in 45 previously hospitalised adults with PCS. Pain intensity was assessed using the visual analogue scale and PPT was assessed by algometry. Health status included fatigue, dyspnoea, anxiety, depression, quality of life, functionality, frailty, physical activity, muscle quality, muscle strength, physical performance, and functional capacity. Analyses were conducted using SPSS v30.0. **Results**: Participants showed moderate pain intensity and variability in PPT, with a significant inverse association between both measures. Bivariate analyses showed that higher pain intensity and lower PPT were associated with worse physical, psychological, and functional outcomes. In regression analyses, pain intensity was associated with sex, length of hospital stay, PPT, and quality of life; PPT was associated with sex, pain intensity, and grip strength. Model explanatory capacity varied, and some models were not statistically significant. **Conclusions**: Subjects with PCS exhibited moderate pain intensity and variability in pain sensitivity, with an association between subjective and objective pain measures. Pain measures were associated with multiple health domains at the bivariate level, while regression analyses identified a limited number of associations with variable explanatory capacity. These findings support comprehensive pain assessment in PCS.

## 1. Introduction

Post-COVID-19 syndrome (PCS) is defined by the National Institute for Health and Care Excellence (NICE) as *the presence of signs and symptoms that develop during or following a COVID-19 infection, persist for more than 12 weeks, and cannot be explained by an alternative diagnosis* [1]. This clinical condition is complex and heterogeneous. It affects multiple organ systems and manifests notably by pain [2,3,4]. Pain is a particularly noteworthy persistent symptom due to its high prevalence (approximately 42%) and its potentially incapacitating nature, underscoring the need for a more detailed characterisation in this population [5].

Pain in PCS may have generalised, musculoskeletal, or neuropathic forms. Its prevalence is higher among subjects who were hospitalised during the acute phase of COVID-19 [6,7,8]. To assess pain exhaustively, it is important to consider both subjective experience, usually assessed by measuring pain intensity with the visual analogue scale (VAS) [9], and objective pain sensitivity, assessed through the pressure pain threshold (PPT) using algometry [10]. These complementary measures provide a comprehensive assessment of pain [9,10].

The presence of pain can influence and be influenced by other health outcomes, such as fatigue, dyspnoea, anxiety, depression, functionality, frailty, quality of life, physical activity, muscular strength, and muscle quality [11,12,13]. These interactions highlight the multidimensional nature of pain, which involves not only nociception and sensory components but also psychological, cognitive, and functional factors [14,15].

However, within the context of PCS, the relationships between pain and these health domains have not been thoroughly explored [16]. Most studies have focused on isolated symptoms, without examining pain from a global perspective that considers its interactions with other aspects of health [7]. This gap emphasises the need for research that applies a multidimensional approach, combining subjective and objective measures of pain and exploring its associations with broader health outcomes.

We hypothesised that pain intensity would be associated with PPT. In addition, we expected both pain intensity and PPT to be associated with outcomes across psychological, functional, and physical health domains. Consequently, the aims of this study were to describe pain in subjects with PCS by assessing pain intensity and PPT; and to examine the associations between these measures and their relationships with descriptive characteristics and health status outcomes using both bivariate and regression analyses. The health status outcomes were fatigue, dyspnoea, anxiety and depression, quality of life, functionality, frailty, physical activity, muscle quality, muscle strength, physical performance, and functional capacity.

## 2. Materials and Methods

### 2.1. Design

This cross-sectional observational study was conducted at the Faculty of Health Sciences, University of Granada, Spain. Subjects’ assessments were performed from March to June 2024, involving a cohort of hospitalised subjects since March 2020. All procedures were carried out in accordance with the Strengthening the Reporting of Observational Studies in Epidemiology (STROBE) guidelines and checklist [17].

### 2.2. Ethical Aspects

This study was conducted in accordance with the principles of the Declaration of Helsinki (1975, revised in 2013). The study protocol was approved by the Provincial Research Ethics Committee of Granada (code: 11/21; date: 21 December 2021).

Prior to participation, all subjects received detailed written information about the study objectives, procedures, potential risks, and data handling, including assurances of confidentiality and anonymity. Written informed consent was obtained from all participants before enrolment.

### 2.3. Subjects

The study recruited subjects from Virgen de las Nieves University Hospital in Granada, Spain. Researchers from the University of Granada provided the Pulmonology Department with predefined inclusion criteria. Using these criteria, the pulmonologists identified subjects who had been hospitalised for COVID-19 from the start of the pandemic until the recruitment period and who, according to their medical records, appeared to meet the preliminary eligibility requirements.

PCS has been defined by the NICE as *the presence of signs and symptoms that develop during or following a confirmed SARS-CoV-2 infection, persist for more than 12 weeks, and cannot be explained by an alternative diagnosis* [1]. PCS diagnosis was established in routine clinical practice by specialist pulmonologists based on patients’ medical records and follow-up information. This classification was subsequently verified by the research team during a structured telephone screening and eligibility confirmation prior to enrolment.

After obtaining prior consent, the clinicians shared the contact details of potential subjects with the research team. All listed subjects were then contacted by telephone. During the call, the research team confirmed that the inclusion criteria were still met, clarified any questions regarding the study, explained its aims and procedures in detail, and verified the subject’s willingness to take part. Subjects who agreed to participate were scheduled for an in-person evaluation.

Inclusion criteria were: (1) being aged 18–80 years; (2) a diagnosis of PCS according to the NICE criteria described above; (3) confirmed SARS-CoV-2 infection by Reverse Transcription-Polymerase Chain Reaction (RT-PCR) or positive rapid antigen test; (4) being clinically stable at recruitment; (5) for women, not being pregnant or breastfeeding; (6) no major surgical procedures within the last six months that could limit participation; (7) willingness to attend scheduled assessment sessions; and (8) absence of medical conditions that could interfere with study involvement. These included uncontrolled cardiovascular diseases (e.g., hypertension, arrhythmias), poorly controlled chronic pulmonary disorders (e.g., asthma, COPD, pulmonary hypertension, active pneumonia), severe musculoskeletal or orthopaedic problems (e.g., recent fractures, significant mobility limitations), neurological or cognitive impairments affecting cooperation, major sensory deficits, acute infections, active cancer or malignancy in the past five years, or any other condition judged by the clinical team as having the potential to worsen during the study.

Subjects who developed an acute medical event or condition (e.g., acute illness, fracture) after initial inclusion but before assessment were excluded.

### 2.4. Data Collection

Initial contact with subjects was made by telephone to provide a detailed explanation of the study and to arrange an in-person evaluation. These evaluations took place at the Department of Physiotherapy, Faculty of Health Sciences, University of Granada, and were conducted by two physiotherapists with specific training and extensive experience in the study procedures. To ensure consistency in data collection, both assessors underwent a standardisation process before participant recruitment, including joint training sessions, protocol review, practice assessments, and agreement on participant instructions, assessment timing, participant positioning, and measurement procedures. Formal inter-rater reliability testing was not performed.

All information obtained during the assessments was systematically recorded in an Excel database. Appropriate measures were implemented to safeguard the confidentiality and security of the data throughout the process.

### 2.5. Study Variables

The variables evaluated in this study included descriptive characteristics of the subjects (age, body mass index, sex, World Health Organization classification, length of hospital and intensive care unit stay, and comorbidities), pain (pain intensity and PPT), and health status (fatigue, dyspnoea, anxiety and depression, quality of life, functionality, frailty, physical activity, muscle quality, muscle strength, physical performance, and functional capacity).

#### 2.5.1. Descriptive Characteristics of the Subjects

Age, sex, World Health Organization (WHO) classification, as well as length of hospital and intensive care unit (ICU) stay were recorded on the assessment day using a standardised form. The information was obtained through questions asked by the assessor and answers provided directly by the participant. Length of ICU stay was recorded as the total number of days spent in the ICU during the acute phase of COVID-19. This variable was analysed at cohort level. Subjects not admitted to the ICU (i.e., non-severe cases) were assigned a value of 0 days. Therefore, length of ICU stay should be interpreted as a zero-inflated healthcare utilisation variable.

Body mass index (BMI) was assessed using the bioelectrical impedance analyser (TANITA SC-240MA, Tanita Corporation, Tokyo, Japan).

Comorbidities were evaluated using the Charlson Comorbidity Index (CCI) [18]. This index comprises 19 medical conditions, each assigned a weight from 1 to 6 according to its influence on prognosis. Participants reported whether they had been diagnosed with each condition, and the corresponding scores were added up to obtain a total value. An additional point was included for every decade of age beyond 50 years. Comorbidity levels were classified as follows: none (0–1 points), low (2 points), or high (≥3 points) [19,20].

#### 2.5.2. Pain

Pain intensity was measured using the VAS, a widely used instrument for quantifying subjective pain perception. It consists of a 100-millimetre horizontal line anchored by two descriptors representing the extremes of pain: “no pain” and “worst imaginable pain.” Participants mark the point on the line that best reflects their current pain level, and the distance in millimetres from the left endpoint is recorded as the numerical value of pain intensity [21].

PPT was collected using algometry to evaluate participants’ pain sensitivity. A digital algometer (Force One FPIX 50, Wagner Instruments, Riverside, CT, USA) was used. This device has a pistol-like shape, featuring a pressure sensor at the tip and a display unit that shows the recorded values. Measurements were performed with participants seated. The examiner applied pressure with the algometer at a constant rate of approximately 1 kilogram (kg)/second (s) on predefined anatomical sites until the participant reported that the sensation changed from pressure to the first perception of discomfort. At that point, the pressure was stopped, and the corresponding value (kg) was recorded. Assessments were conducted on five bilateral sites, alternating between the right and left sides of the body: (1) thumbnail phalanx; (2) tendon of the gracilis muscle at the knee; (3) second rib; (4) supraspinatus muscle; and (5) middle portion of the trapezius muscle, between the superior angle of the scapula and the spine. Each site was tested three times, with a one-minute interval between consecutive measurements. The mean of the three trials was calculated for each site, followed by the mean between sides for each point, and finally the total average PPT [22,23].

#### 2.5.3. Health Status

Fatigue was examined using the Fatigue Severity Scale (FSS), a validated instrument designed to evaluate fatigue severity. The scale includes nine self-administered items rated on a 7-point Likert scale whose total score can range from 9 to 63 points. A total score of 36 or above is considered indicative of clinically relevant fatigue [24,25].

Dyspnoea was evaluated using the Dyspnoea-12 questionnaire, a validated 12-item instrument rated on a 4-point Likert scale (0 = none, 1 = mild, 2 = moderate, 3 = severe). This tool assesses the overall severity of dyspnoea and includes two subscales: physical and emotional. The total score ranges from 0 to 36, with higher scores indicating greater dyspnoea severity [26,27].

Anxiety and depression were assessed using the Hospital Anxiety and Depression Scale (HADS), a widely applied instrument for evaluating these symptoms in the general population. The scale includes 14 items divided into two subscales: anxiety and depression. Each item is scored from 0 (never) to 3 (almost all the time). A total score of 13 points or higher is considered indicative of clinically significant anxiety or depression [28,29].

Quality of life was measured using the European Quality of Life-5 Dimensions questionnaire (EQ-5D). The first section evaluates five dimensions: mobility, self-care, usual activities, pain/discomfort, and anxiety/depression. Each dimension is scored from 1 (no problems) to 3 (extreme problems). These scores are then converted into a summary index ranging from 0 to 1, where 0 represents a health state equivalent to death and 1 represents perfect health. The second section is a VAS where participants rate their overall health on the day of assessment from 0 (worst health) to 100 (best health) [30].

Functionality was analysed using the Post-COVID-19 Functional Status Scale (PCFS), a validated instrument designed to evaluate functional limitations in subjects who currently have or have previously had COVID-19. The scale categorises participants into five levels of functional impairment: 0 = no functional limitations, 1 = symptoms present without significant limitations, 2 = mild limitations, 3 = moderate limitations, and 4 = severe limitations [31,32].

Frailty was recorded using the Clinical Frailty Scale (CFS). This scale evaluates the functional status of participants, with scores ranging from 0 to 9. A score of 0 indicates optimal functional status, while a score of 9 corresponds to terminal illness. Higher scores reflect greater frailty and more severe functional limitations [33,34].

Physical activity was collected using the short form of the International Physical Activity Questionnaire (IPAQ), a standardised and widely validated tool for measuring self-reported physical activity in adults. This version consists of seven items that collect information on the frequency and duration of walking, moderate-intensity, and vigorous-intensity activities carried out over the previous seven days, as well as the typical amount of time spent sitting during a workday [35].

Muscle quality was evaluated with the Muscle Quality Index (MQI), expressed in watts (W) and calculated according to the following formula: MQI = ((L − 0.5) × body weight × g × 5)/T sit-to-stand, where “L” represents leg length, 0.5 metres (m) corresponds to the chair height (50 centimetres), “g” is the gravitational acceleration (9.8 m/s^2^), and “T” denotes the time required to complete the five-times sit-to-stand test. Leg length was determined as the sum of the upper and lower segments: the upper leg measured in standing position from the inguinal fold to the superior pole of the patella along the anterior thigh, and the lower leg measured in a seated position from the lateral femoral condyle to the heel along the lateral side of the leg. Body weight (in kg) was obtained using a bioelectrical impedance scale (TANITA SC-240MA, Tanita Corporation, Tokyo, Japan). For the five-times sit-to-stand test, participants were instructed to stand up and sit down five times as quickly as possible with their arms crossed over their chest. Timing started when the participant was seated and ended upon completion of the fifth stand. Higher MQI values indicate better muscle quality [36,37].

Muscle strength was assessed using dynamometry. Upper limb grip strength was measured with a digital handgrip dynamometer (Takei TKK 5401, Takei Scientific Instruments Co., Ltd., Niigata, Japan). Participants performed three trials with their dominant hand, allowing one minute of rest between attempts. They were seated with their arm alongside the torso without rotation, their elbow bent at 90°, and their wrist in a neutral position. Participants were instructed to squeeze the dynamometer with maximal effort for each trial. The highest value among the three measurements was recorded as the final score [38].

Lower limb strength was recorded using a portable hand-held dynamometer (Lafayette Instrument 01165A, Lafayette Instrument Company, Lafayette, IN, USA), following the method described by Mentiplay et al. [39] to measure isometric strength of the knee extensors and hip flexors. To measure hip flexor strength, participants were seated with hips and knees flexed at 90°, legs hanging freely, hands resting on the table edges, and the trunk stabilised. The dynamometer was placed on the anterior thigh, just above the knee. Knee extensor strength was tested in the same seated position, with the dynamometer applied to the front of the tibia, just above the ankle. During both tests, the assessor provided verbal encouragement to ensure maximal effort. Each muscle group was tested twice for 3–5 s, after a submaximal familiarisation trial, and the highest value was taken as the final measurement.

Physical performance was quantified using the Short Physical Performance Battery (SPPB), which consists of three components: standing balance (scored 0–4), 4 m gait speed (0–4), and the five-times chair stand test (0–4). The total score ranges from 0 to 12, where 0 indicates the lowest level of physical performance and 12 indicates the highest. A total score below 10 points is considered to denote poor physical function, frailty, and an increased risk of falls [40].

Functional capacity was assessed using the 2-Minute Walk Test (2MWT). Participants were asked to walk as far as possible for two minutes along a marked corridor at a self-selected pace, with the possibility to slow down or stop if needed. The total distance walked, measured in metres, was recorded as the outcome of the test [41].

### 2.6. Statistical Analysis

Data analysis was conducted using the IBM Statistical Package for the Social Sciences (SPSS) Statistics, version 30.0 (IBM Corp., Chicago, IL, USA). The software is commercially available and its source code is not publicly accessible. The distribution of continuous variables was evaluated using both visual inspection (histograms and Q-Q plots) and the Kolmogorov–Smirnov test.

Continuous variables were summarised as the mean ± standard deviation (SD) when normally distributed (*p* > 0.05) and as the median with interquartile range (IQR) when the distribution deviated from normality. Categorical variables were presented as absolute and relative frequencies (*n*, %). Statistical significance was set at *p* < 0.05, with 95% confidence intervals (CI) reported where appropriate.

No missing data were identified in the final dataset. Therefore, all analyses were conducted using complete-case analysis.

Associations between pain, descriptive characteristics of the subjects, and health status were examined using correlation analyses for continuous variables. The choice of correlation coefficient (i.e., Pearson’s or Spearman’s) was determined by the distribution of the data. Spearman’s rank correlation was used as the non-parametric alternative for data not meeting the assumptions for Pearson’s correlation. Pearson’s correlation coefficients were interpreted according to the following scale: 0–0.19, none to slight; 0.20–0.39, low; 0.40–0.69, moderate; 0.70–0.89, high; 0.90–1.00, very high [42]. Given the exploratory nature of the correlation analyses and the pre-specified selection of outcomes, no formal correction for multiple comparisons was applied. Therefore, results should be interpreted considering the potential inflation of Type I error.

The consistency of PPT across anatomical sites was assessed using a two-way mixed-effects intraclass correlation coefficient (ICC)—absolute agreement, based on average measures.

In addition, linear regression analyses were conducted to examine factors associated with pain intensity and PPT. Separate models were developed for each dependent variable (i.e., pain intensity and PPT). Depending on the number of predictors included, both simple and multiple linear regression models were developed. Independent variables were grouped into three blocks: (1) descriptive characteristics of the subjects, (2) the alternative pain measure (i.e., PPT in the pain intensity model and pain intensity in the PPT model), and (3) health status variables (dyspnoea, anxiety and depression, quality of life, frailty, physical activity, muscle quality, muscle strength, physical performance, and functional capacity). Given the cross-sectional design and the absence of a predefined causal direction between pain intensity and PPT, simple linear regression models were additionally performed in an exploratory manner. In these models, all pain measures were included as both dependent and independent variables to assess the robustness and consistency of their association. These analyses were conducted within an exploratory and hypothesis-generating framework and were not specifically powered for multivariable modelling; therefore, the possibility of model overfitting and limited stability of regression estimates cannot be excluded. For each model, unstandardised (B) and standardised (β) coefficients, standard errors (SE), *p*-values, and 95% confidence intervals were reported. Model fit was assessed using R^2^ and adjusted R^2^. The magnitude of standardized β coefficients was interpreted as small (0.10), moderate (0.30), or large (0.50) [43,44]. Assumptions of linearity, normality of residuals (explored with the Kolmogorov–Smirnov test and graphical methods: histograms and Q-Q plots), and homoscedasticity were checked, and multicollinearity was assessed using tolerance and variance inflation factor (VIF).

### 2.7. Sample Size

The required sample size was determined using G*Power, version 3.1 (Heinrich Heine University, Düsseldorf, Germany) for a two-tailed correlation analysis between pain, descriptive characteristics of the subjects, and health status. The analysis was conducted with a significance level of α = 0.05 and a statistical power of 80% (1-β = 0.80). No robust prior evidence or pilot data were available to estimate the expected magnitude of the associations in subjects with PCS. Therefore, a moderate effect size (r = 0.40) was assumed according to Cohen’s conventional benchmarks for correlation coefficients [44]. This value was selected following methodological recommendations for sample size planning when empirical effect size estimates are unavailable [45,46]. The calculation indicated that 47 subjects would be required to detect correlations of this magnitude with the specified statistical power.

However, the final sample comprised 45 participants. Therefore, a post hoc power analysis was performed, yielding an achieved statistical power of 79.3%, slightly below the predefined threshold of 80%.

This sample size calculation was based on correlation analyses and was not specifically powered for the linear regression analyses conducted in the study.

## 3. Results

**Figure 1:**
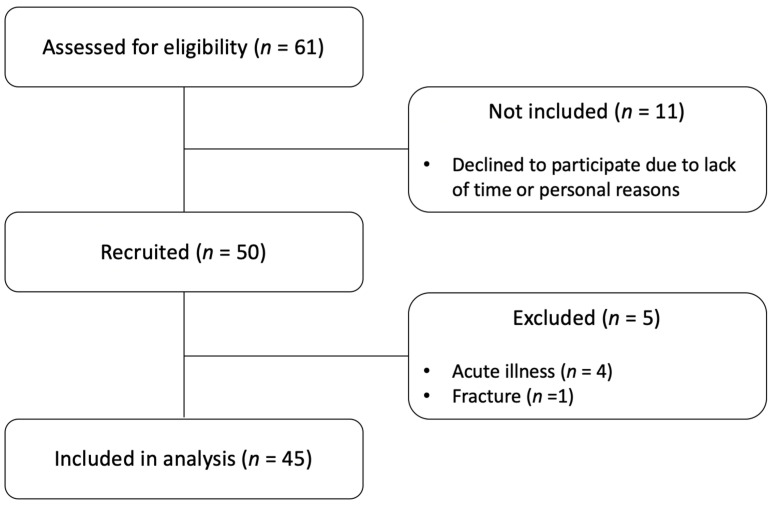
Flow diagram.

Out of 61 eligible subjects, 11 declined to participate due to lack of time or changes in their personal situation. Of the 50 subjects initially recruited, five were excluded prior to assessment due to acute illness (*n* = 4) or fracture (*n* = 1). Consequently, the final sample included 45 participants (Figure 1).

### 3.1. Descriptive Characteristics of the Subjects

Table 1 presents the descriptive characteristics of subjects. The mean age of the sample was 58.5 ± 10.9 years, and the BMI was 30.6 ± 5.7 kg/m^2^. More than half of the participants were men (57.8%). According to the WHO classification, 57.8% of the subjects had experienced a severe form of COVID-19 during the acute phase of the disease, whereas 42.2% were classified as critical cases requiring admission to the ICU. The median length of hospital stay was 15.0 days (IQR = 20.0). Length of ICU stay showed a highly skewed and zero-inflated distribution, with a median of 0.0 days (IQR = 13.0), reflecting the large proportion of non-ICU admissions and the variability among ICU-admitted subjects. This explains the median value of 0 days despite ICU admission in 42.2% of participants. The mean CCI score was 2.84 ± 2.94, indicating a comorbidity level ranging from low to high among participants.

A detailed breakdown of normality results for each variable is available in Supplementary Materials (Table S1).

### 3.2. Pain

Table 2 summarises pain intensity and PPT. Participants reported moderate pain intensity. PPT values differed by anatomical site, with the lowest thresholds observed in the second rib and gracilis muscle, and the highest found in the thumbnail phalanx. The supraspinatus and trapezius muscles exhibited intermediate thresholds. The overall mean PPT across all sites was 3.94 ± 2.21 kg.

The consistency of PPT across anatomical sites showed excellent reliability (ICC = 0.950; 95% CI: 0.907–0.973; *p* < 0.001).

A detailed breakdown of normality results for each variable is available in Supplementary Materials (Table S1).

### 3.3. Health Status

Table 3 summarises participants’ health status. Most participants exhibited relevant fatigue, with median scores exceeding the established cutoff value of 36 points. Dyspnoea was more marked in the physical than in the emotional domain. Anxiety and depression scores indicated that participants were above the clinical thresholds for both conditions. Participants reported a moderate perception of their overall health-related quality of life. Functional status and frailty assessments suggested mostly mild functional limitations and very mild frailty. Although only light-intensity physical activity was reported, the total weekly energy expenditure was 693.0 metabolic equivalents of task (MET)-min/week (IQR = 1287.0), placing participants in the moderate activity category. Muscle quality had a median of 109.56 (IQR = 128.63) W, and muscle strength was relatively similar across upper and lower limbs. Physical performance was below the recommended threshold, consistent with reduced functional capacity and a potential increased risk of falls.

A detailed breakdown of normality results for each variable is available in Supplementary Materials (Table S1).

### 3.4. Correlation Analysis

Table 4 shows the correlation analysis of pain intensity with descriptive characteristics of the subjects, PPT, and health status.

Regarding the descriptive characteristics of the subjects, pain intensity showed no significant associations with age, BMI, length of hospital or ICU stay, or comorbidities.

With respect to PPT, a negative and significant correlation with pain intensity was observed (r = −0.453 moderate; *p* = 0.003). Higher pain intensity values were associated with lower PPT values.

Concerning health status, pain intensity was positively associated with fatigue (rho = 0.439 moderate; *p* = 0.003), total dyspnoea (r = 0.334 low; *p* = 0.025), and the physical domain of dyspnoea (r = 0.362 low; *p* = 0.015). It was also positively associated with anxiety (r = 0.645 moderate; *p* < 0.001) and depression (r = 0.605 moderate; *p* < 0.001), functionality (r = 0.394 low; *p* = 0.007), and frailty (r = 0.371 low; *p* = 0.012). Higher pain intensity was associated with greater fatigue, dyspnoea, anxiety, depression, functional limitations, and frailty in patients with PCS. In addition, pain intensity showed negative correlations with quality of life, both in the EQ-5D index (r = −0.692 moderate; *p* < 0.001) and the EQ-5D VAS (r = −0.386 low; *p* = 0.009), and with muscle quality (rho = −0.357 low; *p* = 0.041), upper limb grip strength (r = −0.376 low; *p* = 0.017), physical performance (r = −0.420 moderate; *p* = 0.013), and functional capacity (rho = −0.374 low; *p* = 0.042). Higher pain intensity was associated with poorer quality of life, lower muscle quality, reduced upper limb grip strength, poorer physical performance, and diminished functional capacity. No significant associations were observed between pain intensity and physical activity or lower limb strength.

Table 5 shows the correlation analysis of PPT with descriptive characteristics of the subjects, pain intensity, and health status.

Exploring the descriptive characteristics of the subjects, PPT did not show significant associations with age, BMI, length of hospital or ICU stay, or comorbidities.

The analysis of the relationship between pain intensity and PPT revealed a negative and significant correlation (r = −0.453 moderate; *p* = 0.003). Higher PPT was associated with lower perceived pain intensity.

When examining health status, PPT was positively associated with quality of life measured by the EQ-5D index (r = 0.329 low; *p* = 0.033), muscle quality (rho = 0.685 moderate; *p* < 0.001), upper limb grip strength (r = 0.711 high; *p* < 0.001), lower limb strength (hip flexor: r = 0.611 moderate; *p* < 0.001; and knee extensor: r = 0.448 moderate; *p* = 0.008), physical performance (r = 0.397 low; *p* = 0.020), and functional capacity (rho = 0.565 moderate; *p* = 0.001). Higher PPT, and therefore lower pain sensitivity, was associated with better quality of life, greater muscle quality, strength, and physical performance, as well as higher functional capacity in subjects with PCS. In addition, PPT showed negative correlations with fatigue (rho = −0.480 moderate; *p* = 0.001), dyspnoea, both in the total score (r = −0.400 moderate; *p* = 0.009) and in the physical (r = −0.379 low; *p* = 0.013) and emotional domains (r = −0.390 low; *p* = 0.011), anxiety (r = −0.412 moderate; *p* = 0.007), depression (r = −0.480 moderate; *p* = 0.001), functionality (r = −0.349 low; *p* = 0.023), and frailty (r = −0.373 low; *p* = 0.015). Higher PPT was associated with lower fatigue, dyspnoea, anxiety, depression, functional limitations, and frailty. Finally, no significant associations were observed between PPT and physical activity.

### 3.5. Linear Regression Analyses

Table 6 presents the linear regression analyses for pain intensity.

In the model including descriptive characteristics (multiple linear regression), the overall model explained 30.0% of the variance (R^2^ = 0.300; adjusted R^2^ = 0.167), but did not reach statistical significance (*p* = 0.051). Sex (β = 0.403 moderate; 95% CI: 0.751 to 4.639; *p* = 0.008) and length of hospital stay (β = −0.654 large; 95% CI: −0.191 to −0.033; *p* = 0.007) showed statistically significant coefficients. Yet, these findings should be interpreted with caution because the overall model was not statistically significant.

In the model including PPT (simple linear regression), a statistically significant association was observed (β = −0.453 moderate; 95% CI: −1.099 to −0.251; *p* = 0.003). This model explained 20.6% of the variance (R^2^ = 0.206; adjusted R^2^ = 0.186) and was statistically significant (*p* = 0.003).

In the model including health status variables (multiple linear regression), only the EQ-5D index was significantly associated with pain intensity (β = −0.901 large; 95% CI: −15.938 to −2.759; *p* = 0.009). The model explained 66.0% of the variance (R^2^ = 0.660; adjusted R^2^ = 0.411) and was statistically significant (*p* = 0.041).

No statistically significant associations were identified for the remaining variables across models. No relevant multicollinearity issues were detected (all VIF < 5).

A detailed analysis of the normality of standardised residuals for each model is available in Supplementary Materials (Table S2).

Table 7 presents the linear regression analyses for PPT.

In the model including descriptive characteristics (multiple linear regression), the overall model explained 23.8% of the variance (R^2^ = 0.238; adjusted R^2^ = 0.081) but did not reach statistical significance (*p* = 0.195). Although sex showed a statistically significant coefficient (β = −0.503 large; 95% CI: −3.680 to −0.808; *p* = 0.003), this finding should be interpreted with caution because the overall model was not statistically significant.

In the model including pain intensity (simple linear regression), a statistically significant association was observed (β = −0.453 moderate; 95% CI: −0.496 to −0.113; *p* = 0.003). This model explained 20.6% of the variance (R^2^ = 0.206; adjusted R^2^ = 0.186) and was statistically significant (*p* = 0.003).

In the model including health status variables (multiple linear regression), the overall model explained 54.3% of the variance (R^2^ = 0.543; adjusted R^2^ = 0.208) but did not reach statistical significance (*p* = 0.190). Although upper limb grip strength showed a statistically significant association with PPT (β = 0.714 large; 95% CI: 0.006 to 0.237; *p* = 0.041), this result should be interpreted with caution given the non-significant overall model.

No statistically significant associations were identified for the remaining variables across models. No relevant multicollinearity issues were detected (all VIF < 5).

A detailed analysis of the normality of standardized residuals for each model is available in Supplementary Materials (Table S2).

## 4. Discussion

The aims of this study were to describe pain in subjects with PCS using both subjective (pain intensity) and objective (PPT) measures; and to explore the associations between these measures and their relationships with the descriptive characteristics and health status of this population, including both bivariate and regression analyses.

Firstly, our results showed that participants experienced moderate pain intensity, as measured by the VAS. This is consistent with previous studies such as those by Fernández-de-las-Peñas et al. [47]. These authors reported that, although pain intensity was generally moderate, many subjects who were healthy prior to infection developed pain following COVID-19, which may be a risk factor for the development of chronic pain if appropriate management strategies are not applied. Similarly, Bileviciute-Ljungar et al. [48] reported that pain, in addition to being a persistent symptom in PCS, could remain at clinically relevant levels for several months. These results underscore the importance of assessing and monitoring pain in this population to ensure timely management and prevent long-term consequences.

In addition to subjective pain, our study found that objective pain sensitivity varied across anatomical sites, with the lowest PPT in the second rib and the highest in the thumbnail phalanx. Despite these differences, the overall pattern showed a coherent distribution of pain sensitivity in subjects with PCS, emphasising the value of combining subjective and objective measures for a comprehensive assessment [49].

Regarding associations with descriptive characteristics, neither the bivariate nor the regression analyses showed a clear or consistent pattern for pain intensity. In the correlation analysis, pain intensity was not associated with the descriptive characteristics of the subjects. In the regression analyses, sex and length of hospital stay showed statistically significant coefficients within the model but the overall model did not reach statistical significance. This shows that these variables have limited explanatory capacity. Therefore, these associations should be interpreted as exploratory and with caution. This is consistent with the study by Fernández-de-las-Peñas et al. [50], who reported that basic demographic variables had a limited ability to explain the variability in pain experience in PCS, supporting the idea that persistent pain following SARS-CoV-2 infection may involve mechanisms beyond simple descriptive characteristics.

In contrast, a more consistent pattern emerged in the association between pain intensity and PPT. The bivariate analysis showed that higher pain intensity was associated with lower PPT. The association remained significant in the regression analysis. This finding supports the coherence between subjective and objective pain measures. It is consistent with greater pain sensitivity in subjects reporting higher pain intensity, which may be related to mechanisms such as central sensitization [51]. Nevertheless, given the cross-sectional design, these associations should not be interpreted as causal relationships.

With respect to health status, the bivariate analysis showed that pain intensity was associated with increased fatigue, dyspnoea, anxiety, depression, functional limitations, and frailty, as well as poorer quality of life, lower muscle quality, reduced upper limb grip strength, poorer physical performance, and diminished functional capacity. However, when these variables were analysed together in the regression analysis, only quality of life remained significantly associated with pain intensity. This suggests that many of the observed bivariate associations may be interrelated, with quality of life potentially capturing a broader dimension of the overall health burden in PCS. These findings align with studies describing PCS as a condition characterised by interrelated symptoms [52,53]. No associations were observed with physical activity levels or lower limb strength in either analysis. This suggests that pain perception may be more closely linked to overall health status than to isolated peripheral physical impairments [54,55].

A similar pattern was observed for PPT. In the bivariate analysis, PPT showed no association with the descriptive characteristics of the subjects. In the regression analysis, although sex showed a statistically significant coefficient within the model, the overall model was not statistically significant, indicating limited explanatory capacity of these variables. These findings are consistent with previous research [56]. Importantly, both the bivariate and the regression analyses showed that higher PPT values, reflecting lower pain sensitivity, were associated with lower pain intensity, reinforcing the coherence between measures [51]. Additionally, higher PPT values were generally associated with more favourable health status indicators in the bivariate analysis; however, these associations were not consistently observed in the regression analyses. In line with our findings, evidence in other populations suggests that pain sensitivity may be related to the overall symptom burden, including fatigue, psychological distress, and other related symptoms [57].

Overall, these results emphasise that integrating subjective and objective measures of pain provides a comprehensive understanding of the multidimensional burden of PCS. They also highlight the importance of considering the combined interpretation of bivariate and regression analyses to avoid overinterpretation of isolated associations.

### 4.1. Limitations and Strengths

The study has several limitations that should be considered. Its cross-sectional design prevents establishing causal relationships between pain measures and health outcomes.

The final sample size (*n* = 45) was slightly lower than that estimated in the a priori calculation (*n* = 47). Nevertheless, this small difference is unlikely to have meaningfully affected the results, as the post hoc power analysis indicated an achieved statistical power of 79.3%, very close to the predefined threshold of 80%.

The sample was drawn from a single hospital and was restricted to patients with a history of hospitalisation for COVID-19. This may have introduced selection bias and limited the external validity of the findings. This applies particularly to individuals with milder or non-hospitalised forms of PCS who may present different clinical characteristics and symptom severity. However, this cohort is a clinically relevant subgroup characterised by a higher symptom burden and greater functional impairment. This makes it particularly appropriate for the detailed characterisation of multidimensional aspects of pain in PCS.

Additionally, some variables were self-reported, making them susceptible to recall or response bias. Although PPT provides objective information on pain sensitivity, it does not allow a comprehensive assessment of the underlying mechanisms of pain, and complementary evaluations would be necessary in future studies.

All assessments were conducted by trained physiotherapists following a standardised protocol. However, formal inter-rater reliability was not evaluated. Hence, some degree of measurement variability between assessors cannot be completely excluded.

From a statistical perspective, although the a priori sample size calculation was based on correlation analyses, the study also included linear regression analyses that were not specifically powered for multivariable modelling. In this context, considering the relatively small sample size and the number of predictors included in some models, the possibility of model overfitting and reduced stability of regression estimates cannot be excluded. These analyses were conducted within an exploratory framework, and the findings should be interpreted with caution.

Finally, no correction for multiple comparisons was applied in the correlation analyses, which may increase the risk of Type I error; accordingly, the results should also be considered as exploratory rather than confirmatory.

Despite these limitations, this study also presents several strengths. Notably, it provides a comprehensive assessment of pain in subjects with PCS, combining subjective and objective measures.

Furthermore, multiple health domains were evaluated (i.e., fatigue, dyspnoea, anxiety, depression, functionality, frailty, quality of life, physical activity, muscle quality, strength, physical performance, and functional capacity). This enabled a detailed analysis of the interrelationships between pain and other aspects of health status.

Finally, all assessments were conducted by trained and experienced physiotherapists following standardised procedures, and the study adhered to ethical standards and the STROBE reporting guidelines.

### 4.2. Clinical Implications

The presence of moderate pain intensity and the variability of PPT in subjects with PCS highlights the need for a multidimensional assessment of pain, combining both subjective and objective measures. Clinicians should also recognise that pain in PCS is closely associated with fatigue, dyspnoea, psychological distress, functional limitations, frailty, quality of life, and reduced physical performance. This highlights the importance of an integrated approach to patient management. Pain-targeted interventions may benefit from strategies that include both physical rehabilitation and psychological support, rather than focusing solely on isolated symptoms. Untreated pain can result in a substantial burden of work absenteeism and economic losses, affecting patients and healthcare systems. For this reason, its early detection and management are key. Therefore, it is essential to develop personalised rehabilitation strategies and optimise recovery in this population.

## 5. Conclusions

The findings of this study showed that subjects with PCS exhibited moderate pain intensity and variability in PPT. A moderate relationship was observed between both measures, suggesting an association between subjective pain intensity and objective pain sensitivity.

Bivariate analysis showed that both pain intensity and PPT were associated with higher levels of fatigue, dyspnoea, anxiety, depression, functional limitations, and frailty, as well as lower physical performance and functional capacity. No associations were observed with descriptive characteristics or physical activity.

In regression models (simple and multiple), fewer variables remained associated with pain outcomes. Regarding pain intensity, sex, length of hospital stay, PPT, and quality of life showed significant associations at the variable level. However, the model based on descriptive characteristic did not reach overall statistical significance, and the explanatory capacity varied across models. This suggests that these findings should be interpreted within an exploratory framework.

Regarding PPT, sex, pain intensity, and grip strength were associated with the outcome at the variable level. However, neither the model including descriptive characteristics nor the model including health status variables reached overall statistical significance. In addition, the explanatory capacity again varied across models, reinforcing the exploratory nature of these results.

Overall, these results suggest that pain in PCS may reflect a multidimensional pattern of associations involving subjective and objective measures of pain and broader health status domains. Differences observed between bivariate and regression analyses highlight the importance of considering both approaches jointly.

These findings underscore the need for a comprehensive assessment of pain in this population. Future studies should include larger sample sizes and longitudinal designs to examine changes in pain intensity and PPT over time. They should also further explore underlying pain mechanisms, analyse progression across disease severities, and evaluate the effectiveness of personalised interventions to improve clinical management.

## Figures and Tables

**Table 1:** Descriptive characteristics of subjects.

Variable	Total (*n* = 45)
Age (years)	58.5 ± 10.9
BMI (kg/m^2^)	30.6 ± 5.7
Male sex (*n*, %)	26, 57.8
WHO classification (*n*, %)	
Severe	26, 57.8
Critical	19, 42.2
Length of hospital stay (days)	15.0 (20.0)
Length of ICU stay (days)	0.0 (13.0)
Comorbidities (CCI)	2.84 ± 2.94

Abbreviations. BMI: body mass index; CCI: Charlson Comorbidity Index; ICU: intensive care unit; kg: kilograms; m: metres; *n*: number; WHO: World Health Organization. Note: Continuous variables are expressed as the mean ± standard deviation for normally distributed data or median (interquartile range) for non-normally distributed data. Categorical variables are presented as *n*, %. Length of ICU stay includes all subjects; non-ICU subjects were coded as 0 days.

**Table 2:** Descriptive results for pain in the total sample.

Variable		Total (*n* = 45)
Pain intensity (VAS)		5.09 ± 3.34
PPT (algometry, kg)		
Thumbnail phalanx	Right	5.63 ± 2.74
	Left	5.37 ± 2.73
	Average	5.50 ± 2.72
Gracilis muscle	Right	3.03 ± 1.83
	Left	3.00 ± 1.80
	Average	3.01 ± 1.78
Second rib	Right	2.61 ± 1.77
	Left	2.66 ± 1.87
	Average	2.63 ± 1.80
Supraspinatus muscle	Right	4.32 ± 2.71
	Left	4.25 ± 2.73
	Average	4.29 ± 2.71
Trapezius muscle	Right	4.47 ± 2.96
	Left	4.33 ± 2.87
	Average	4.40 ± 2.89
Total average		3.94 ± 2.21

Abbreviations. kg: kilograms; PPT: pressure pain threshold; VAS: visual analogue scale. Note: Continuous variables are expressed as the mean ± standard deviation for normally distributed data.

**Table 3:** Descriptive results for health status in the total sample.

Variable	Total (*n* = 45)
Fatigue (FSS)	54.0 (28.0)
Dyspnoea (Dyspnoea-12)	
Physical domain	6.51 ± 5.64
Emotional domain	3.53 ± 4.27
Total score	10.04 ± 9.56
Anxiety and depression (HADS)	
Anxiety	9.67 ± 6.63
Depression	7.07 ± 5.43
Total score	16.73 ± 11.42
Quality of life (EQ-5D)	
EQ-5D index	0.48 ± 0.43
EQ-5D VAS	55.78 ± 18.21
Functionality (PCFS)	1.91 ± 1.06
Frailty (CFS)	3.84 ± 1.28
Physical activity (IPAQ, MET-min/week)	
Vigorous activity	0.00 (0.00)
Moderate activity	0.00 (0.00)
Light activity	693.00 (1287.00)
Total activity	693.00 (1287.00)
Muscle quality (MQI, W)	109.56 (128.63)
Muscle strength (dynamometry, kg)	
Upper limb grip strength	27.85 ± 12.82
Hip flexor strength	22.23 ± 9.64
Knee extensor strength	27.13 ± 15.01
Physical performance (SPPB)	8.56 ± 2.38
Functional capacity (2MWT, m)	172.00 (63.60)

Abbreviations. CFS: Clinical Frailty Scale; EQ-5D: European Quality of Life-5 Dimensions questionnaire; FSS: Fatigue Severity Scale; HADS: Hospital Anxiety and Depression Scale; IPAQ: International Physical Activity Questionnaire; kg: kilograms; m: metres; MET: Metabolic Equivalent of Task; min: minutes; MQI: Muscle Quality Index; PCFS: Post-COVID-19 Functional Status Scale; SPPB: Short Physical Performance Battery; VAS: visual analogue scale; W: watts; 2MWT: 2-Minute Walk Test. Note: Continuous variables are expressed as the mean ± standard deviation for normally distributed data or the median (interquartile range) for non-normally distributed data.

**Table 4:** Correlation analysis of pain intensity with descriptive characteristics of subjects, PPT, and health status.

Variable	Total (*n* = 45)		
	Correlation Coefficient		
	Pearson (r)	Spearman (rho)	*p*
**Descriptive characteristics of subjects**			
Age (years)	−0.114	-	0.455
BMI (kg/m^2^)	0.069	-	0.653
Length of hospital stay (days)	-	−0.151	0.321
Length of ICU stay (days)	-	0.141	0.354
Comorbidities (CCI)	−0.038	-	0.804
**PPT (algometry, kg)**			
Total mean	−0.453	-	0.003 *
**Health status**			
Fatigue (FSS)	-	0.439	0.003 *
Dyspnoea (Dyspnoea-12)			
Physical domain	0.362	-	0.015 *
Emotional domain	0.269	-	0.074
Total score	0.334	-	0.025 *
Anxiety and depression (HADS)			
Anxiety	0.645	-	<0.001 **
Depression	0.605	-	<0.001 **
Total score	0.662	-	<0.001 **
Quality of life (EQ-5D)			
EQ-5D index	−0.692	-	<0.001 **
EQ-5D VAS	−0.386	-	0.009 *
Functionality (PCFS)	0.394	-	0.007 *
Frailty (CFS)	0.371	-	0.012 *
Physical activity (IPAQ, MET-min/week)			
Total activity	-	−0.242	0.109
Muscle quality (MQI, W)	-	−0.357	0.041 *
Muscle strength (dynamometry, kg)			
Upper limb grip strength	−0.376	-	0.017 *
Hip flexor strength	−0.272	-	0.109
Knee extensor strength	−0.211	-	0.223
Physical performance (SPPB)	−0.420	-	0.013 *
Functional capacity (2MWT, m)	-	−0.374	0.042 *

Abbreviations. BMI: body mass index; CCI: Charlson Comorbidity Index; CFS: Clinical Frailty Scale; EQ-5D: European Quality of Life-5 Dimensions questionnaire; FSS: Fatigue Severity Scale; HADS: Hospital Anxiety and Depression Scale; ICU: intensive care unit; IPAQ: International Physical Activity Questionnaire; kg: kilograms; m: metres; MET: Metabolic Equivalent of Task; min: minutes; MQI: Muscle Quality Index; PCFS: Post-COVID-19 Functional Status Scale; PPT: pressure pain threshold; SPPB: Short Physical Performance Battery; VAS: visual analogue scale; W: watts; 2MWT: 2-Minute Walk Test. Note: *: *p* < 0.05; **: *p* < 0.001.

**Table 5:** Correlation analysis of PPT with descriptive characteristics of subjects, pain intensity, and health status.

Variable	Total (*n* = 45)		
	Correlation Coefficient		
	Pearson (r)	Spearman (rho)	*p*
**Descriptive characteristics of subjects**			
Age (years)	0.048	-	0.763
BMI (kg/m^2^)	−0.072	-	0.652
Length of hospital stay (days)	-	−0.139	0.381
Length of ICU stay (days)	-	−0.128	0.418
Comorbidities (CCI)	0.037	-	0.817
**Pain intensity (VAS)**	−0.453	-	0.003 *
**Health status**			
Fatigue (FSS)	-	−0.480	0.001 *
Dyspnoea (Dyspnoea-12)			
Physical domain	−0.379	-	0.013 *
Emotional domain	−0.390	-	0.011 *
Total score	−0.400	-	0.009 *
Anxiety and depression (HADS)			
Anxiety	−0.412	-	0.007 *
Depression	−0.480	-	0.001 *
Total score	−0.470	-	0.002 *
Quality of life (EQ-5D)			
EQ-5D index	0.329	-	0.033 *
EQ-5D VAS	0.230	-	0.143
Functionality (PCFS)	−0.349	-	0.023 *
Frailty (CFS)	−0.373	-	0.015 *
Physical activity (IPAQ, MET-min/week)			
Total activity	-	0.070	0.661
Muscle quality (MQI, W)	-	0.685	<0.001 **
Muscle strength (dynamometry, kg)			
Upper limb grip strength	0.711	-	<0.001 **
Hip flexor strength	0.611	-	<0.001 **
Knee extensor strength	0.448	-	0.008 *
Physical performance (SPPB)	0.397	-	0.020 *
Functional capacity (2MWT, m)	-	0.565	0.001 *

Abbreviations. BMI: body mass index; CCI: Charlson Comorbidity Index; CFS: Clinical Frailty Scale; EQ-5D: European Quality of Life-5 Dimensions questionnaire; FSS: Fatigue Severity Scale; HADS: Hospital Anxiety and Depression Scale; ICU: intensive care unit; IPAQ: International Physical Activity Questionnaire; kg: kilograms; m: metres; MET: Metabolic Equivalent of Task; min: minutes; MQI: Muscle Quality Index; PCFS: Post-COVID-19 Functional Status Scale; PPT: pressure pain threshold; SPPB: Short Physical Performance Battery; VAS: visual analogue scale; W: watts; 2MWT: 2-Minute Walk Test. Note: *: *p* < 0.05; **: *p* < 0.001.

**Table 6:** Linear regression analyses of pain intensity (*n* = 45).

Variable	B	SE	β	*p*	95% CI	Tol	VIF
**Descriptive characteristics of subjects**							
Constant	7.421	5.397	-	0.177	−3.515, 18.357	-	-
Age (years)	−0.049	0.063	−0.161	0.438	−0.176, 0.078	0.451	2.216
BMI (kg/m^2^)	0.016	0.086	0.027	0.854	−0.158, 0.190	0.874	1.144
Sex male (*n*, %)	2.695	0.959	0.403	0.008 *	0.751, 4.639	0.917	1.091
WHO classification (*n*, %)	0.028	1.353	0.004	0.984	−2.714, 2.770	0.461	2.170
Length of hospital stay (days)	−0.112	0.039	−0.654	0.007 *	−0.191, −0.033	0.367	2.726
Length of ICU stay (days)	0.117	0.062	0.524	0.069	−0.009, 0.243	0.242	4.133
Comorbidities (CCI)	0.175	0.228	0.154	0.447	−0.287, 0.638	0.469	2.134
*p* = 0.051; R = 0.548; R^2^ = 0.300; Adjusted R^2^ = 0.167; SE = 3.044; DW = 2.214							
**PPT (algometry, kg)**							
Constant	7.491	0.945	-	<0.001 **	5.581, 9.401	-	-
Total mean	−0.675	0.210	−0.453	0.003 *	−1.099, −0.251	1.000	1.000
*p* = 0.003 *; R = 0.453; R^2^ = 0.206; Adjusted R^2^ = 0.186; SE = 2.976; DW = 1.231							
**Health status**							
Constant	16.674	6.079	-	0.015 *	3.716, 29.631	-	-
Dyspnoea (Dyspnoea-12)	−0.104	0.099	−0.295	0.310	−0.314, 0.107	0.288	3.473
Anxiety and depression (HADS)	−0.016	0.102	−0.050	0.876	−0.233, 0.200	0.232	4.306
Quality of life (EQ-5D)							
EQ-5D index	−9.348	3.092	−0.901	0.009 *	−15.938, −2.759	0.256	3.913
EQ-5D VAS	−0.061	0.052	−0.331	0.258	−0.171, 0.049	0.287	3.489
Frailty (CFS) (units)	−0.260	0.767	−0.086	0.739	−1.895, 1.375	0.357	2.803
Physical activity (IPAQ, MET-min/week)	0.000	0.000	0.207	0.304	0.000, 0.001	0.601	1.663
Muscle quality (MQI, W)	0.014	0.012	0.328	0.282	−0.013, 0.041	0.262	3.815
Muscle strength (dynamometry, kg)							
Upper limb grip strength	−0.053	0.072	−0.204	0.469	−0.206, 0.099	0.300	3.335
Knee extensor strength	0.028	0.065	0.126	0.673	−0.110, 0.165	0.266	3.760
Physical performance (SPPB)	−0.293	0.312	−0.212	0.365	−0.959, 0.372	0.443	2.256
Functional capacity (2MWT, m)	0.006	0.017	0.079	0.745	−0.031, 0.395	0.395	2.530
*p* = 0.041 *; R = 0.812; R^2^ = 0.660; Adjusted R^2^ = 0.411; SE = 2.424; DW = 2.043							

Abbreviations. B: unstandardised coefficients; β: standardized Beta coefficients; BMI: body mass index; CCI: Charlson Comorbidity Index; CFS: Clinical Frailty Scale; CI: confidence interval; DW: Durbin-Watson; EQ-5D: European Quality of Life-5 Dimensions questionnaire; HADS: Hospital Anxiety and Depression Scale; ICU: intensive care unit; IPAQ: International Physical Activity Questionnaire; kg: kilograms; m: metres; MET: Metabolic Equivalent of Task; min: minutes; MQI: Muscle Quality Index; *n*: number; PPT: pressure pain threshold; SE: standard error; SPPB: Short Physical Performance Battery; Tol: Tolerance; VAS: visual analogue scale; VIF: variance inflation factor; W: watts; WHO: World Health Organization; 2MWT: 2-Minute Walk Test. Note: *: *p* < 0.05; **: *p* < 0.001.

**Table 7:** Linear regression analyses of the PPT (*n* = 45).

Variable	B	SE	β	*p*	95% CI	Tol	VIF
**Descriptive characteristics of subjects**							
Constant	4.741	3.850	-	0.227	−3.082, 12.565	-	-
Age (years)	−0.013	0.048	−0.064	0.787	−0.111, 0.085	0.404	2.475
BMI (kg/m^2^)	−0.048	0.064	−0.123	0.462	−0.177, 0.082	0.818	1.223
Sex male (*n*, %)	−2.244	0.707	−0.503	0.003 *	−3.680, −0.808	0.893	1.120
WHO classification (*n*, %)	0.588	1.001	0.133	0.561	−1.445, 2.621	0.438	2.284
Length of hospital stay (days)	0.026	0.027	0.236	0.344	−0.029, 0.082	0.371	2.698
Length of ICU stay (days)	−0.038	0.044	−0.267	0.387	−0.127, 0.051	0.242	4.125
Comorbidities (CCI)	0.003	0.167	0.004	0.986	−0.337, 0.343	0.426	2.347
*p* = 0.195; R = 0.488; R^2^ = 0.238; Adjusted R^2^ = 0.081; SE = 2.124; DW = 2.053							
**Pain intensity (VAS)**							
Constant	5.408	0.552	-	<0.001 **	4.293, 6.523	-	-
Total mean	−0.305	0.095	−0.453	0.003 *	−0.496, −0.113	1.000	1.000
*p* = 0.003 *; R = 0.453; R^2^ = 0.206; Adjusted R^2^ = 0.186; SE = 2.000; DW = 1.788							
**Health status**							
Constant	0.038	4.608	-	0.994	−9.783, 9.859	-	-
Dyspnoea (Dyspnoea-12)	−0.016	0.075	−0.071	0.829	−0.176, 0.143	0.288	3.473
Anxiety and depression (HADS)	0.094	0.077	0.443	0.240	−0.070, 0.258	0.232	4.306
Quality of life (EQ-5D)							
EQ-5D index	2.285	2.343	0.337	0.345	−2.709, 7.208	0.256	3.913
EQ-5D VAS	0.017	0.039	0.141	0.672	−0.067, 0.100	0.287	3.489
Frailty (CFS)	−0.460	0.581	−0.231	0.441	−1.699, 0.779	0.357	2.803
Physical activity (IPAQ, MET-min/week)	0.000	0.000	−0.005	0.982	−0.001, 0.001	0.601	1.663
Muscle quality (MQI, W)	0.004	0.009	0.129	0.710	−0.017, 0.024	0.262	3.815
Muscle strength (dynamometry, kg)							
Upper limb grip strength	0.121	0.054	0.714	0.041 *	0.006, 0.237	0.300	3.335
Knee extensor strength	−0.046	0.049	−0.317	0.363	−0.150, 0.058	0.266	3.760
Physical performance (SPPB)	−0.081	0.237	−0.090	0.763	−0.586, 0.423	0.443	2.256
Functional capacity (2MWT, m)	−0.001	0.013	−0.022	0.938	−0.029, 0.027	0.395	2.530
*p* = 0.190; R = 0.737; R^2^ = 0.543; Adjusted R^2^ = 0.208; SE = 1.837; DW = 1.786							

Abbreviations. B: unstandardized coefficients; β: standardized Beta coefficients; BMI: body mass index; CCI: Charlson Comorbidity Index; CFS: Clinical Frailty Scale; CI: confidence interval; DW: Durbin-Watson; EQ-5D: European Quality of Life-5 Dimensions questionnaire; HADS: Hospital Anxiety and Depression Scale; ICU: intensive care unit; IPAQ: International Physical Activity Questionnaire; kg: kilograms; m: metres; MET: Metabolic Equivalent of Task; min: minutes; MQI: Muscle Quality Index; *n*: number; PPT: pressure pain threshold; SE: standard error; SPPB: Short Physical Performance Battery; Tol: Tolerance; VAS: visual analogue scale; VIF: variance inflation factor; W: watts; WHO: World Health Organization; 2MWT: 2-Minute Walk Test. Note: *: *p* < 0.05; **: *p* < 0.001.

## Data Availability

The raw data supporting the conclusions of this article will be made available by the authors on request. The datasets are not publicly available due to privacy, ethical, and data protection requirements, as they contain clinical information about human participants, and their release requires additional safeguards to ensure participant confidentiality and compliance with the ethical approval granted by the relevant committee.

## Supplementary Materials

The following supporting information can be downloaded at: https://www.mdpi.com/article/10.3390/diagnostics16132023/s1. Table S1: Results of the Kolmogorov-Smirnov normality test for continuous variables included in the study. Table S2: Results of the Kolmogorov-Smirnov normality test of standardized residuals.

## Abbreviations

The following abbreviations are used in this manuscript:

BMI	Body mass index
CCI	Charlson Comorbidity Index
CFS	Clinical Frailty Scale
CI	Confidence intervals
EQ-5D	European Quality of Life-5 Dimensions questionnaire
FSS	Fatigue Severity Scale
HADS	Hospital Anxiety and Depression Scale
ICC	Intraclass Correlation Coefficient
ICU	Intensive care unit
IPAQ	International Physical Activity Questionnaire
IQR	Interquartile range
Kg	Kilograms
m	Metres
MET	Metabolic Equivalent of Task
Min	Minutes
MQI	Muscle Quality Index
NICE	National Institute for Health and Care Excellence
PCS	Post-COVID-19 Syndrome
PCFS	Post-COVID-19 Functional Status Scale
PPT	Pressure pain threshold
RT-PCR	Reverse Transcription-Polymerase Chain Reaction
s	Seconds
SD	Standard deviation
SPPB	Short Physical Performance Battery
SPSS	Statistical Package for the Social Sciences
STROBE	Strengthening the Reporting of Observational Studies in Epidemiology
VAS	Visual analogue scale
W	Watts
WHO	World Health Organization
2MWT	2-Minute Walk Test
