# Supplementary material for: Characterising Pain in Post-COVID-19 Syndrome: An Observational Study of Intensity and Sensitivity
Source: Diagnostics (Basel). 2026 Jun 29;16(13):2023. doi: 10.3390/diagnostics16132023 (PMC13359664; doi:10.3390/diagnostics16132023)
Supplement: Supplementary file 1 [file diagnostics-16-02023-s001.zip › diagnostics-4329442-supplementary.pdf]

Supplementary Materials

Table S1: Results of the Kolmogorov-Smirnov normality test for continuous variables included in the study.

Variable	Kolmogorov-Smirnov statistic (D)	p-value	Distribution
**Descriptive characteristics of subjects**			
Age (years)	0.127	0.066	Normal
BMI (kg/m²)	0.097	0.200	Normal
Length of hospital stay (days)	0.211	< 0.001*	Non-normal
Length of ICU stay (days)	0.297	< 0.001*	Non-normal
Comorbidities (CCI)	0.169	0.002*	Normal†
**Pain**			
Pain intensity (VAS)	0.142	0.024*	Normal†
PPT (algometry, kg)			
Thumbnail phalanx			
Right	0.102	0.200	Normal
Left	0.086	0.200	Normal
Average	0.107	0.200	Normal
Gracilis muscle			
Right	0.137	0.058	Normal
Left	0.143	0.033*	Normal†
Average	0.158	0.011*	Normal†
Second rib			
Right	0.156	0.012*	Normal†
Left	0.185	< 0.001*	Normal†
Average	0.172	0.003*	Normal†
Supraspinatus muscle			
Right	0.120	0.142	Normal
Left	0.094	0.200	Normal
Average	0.098	0.200	Normal
Trapezius muscle			
Right	0.163	0.008*	Normal†
Left	0.127	0.092	Normal
Average	0.125	0.108	Normal
Total average	0.089	0.200	Normal
**Health status**			
Fatigue (FSS)	0.237	< 0.001*	Non-normal
Dyspnoea (Dyspnoea-12)			
Physical domain	0.124	0.079	Normal
Emotional domain	0.234	< 0.001*	Normal†
Total score	0.147	0.016*	Normal†
Anxiety and depression (HADS)			
Anxiety	0.137	0.033*	Normal†
Depression	0.151	0.012*	Normal†
Total score	0.115	0.168	Normal
Quality of life (EQ-5D)			
EQ-5D index	0.169	0.002*	Normal†
EQ-5D VAS	0.153	0.010*	Normal†
Functionality (PCFS)	0.178	0.001*	Normal†

Frailty (CFS)	0.229	< 0.001*	Normal†
Physical activity (IPAQ, MET-min/week)			
Vigorous activity	0.513	< 0.001	Non-normal
Moderate activity	0.472	< 0.001	Non-normal
Light activity	0.177	0.001	Non-normal
Total activity	0.220	< 0.001	Non-normal
Muscle quality (MQI, W)	0.215	< 0.001	Non-normal
Muscle strength (dynamometry, kg)			
Upper limb grip strength	0.081	0.200	Normal
Hip flexor strength	0.104	0.200	Normal
Knee extensor strength	0.131	0.139	Normal
Physical performance (SPPB)	0.142	0.078	Normal
Functional capacity (2MWT, m)	0.133	0.008	Non-normal

Abbreviations. BMI: body mass index; CCI: Charlson Comorbidity Index; CFS: Clinical Frailty Scale; EQ-5D: European Quality of Life-5 Dimensions questionnaire; FSS: Fatigue Severity Scale; HADS: Hospital Anxiety and Depression Scale; ICU: intensive care unit; IPAQ: International Physical Activity Questionnaire; kg: kilograms; m: metres; MQI: Muscle Quality Index; PCFS: Post-COVID-19 Functional Status Scale; PPT: pressure pain threshold; SPPB: Short Physical Performance Battery; VAS: visual analogue scale; W: watts; 2MWT: 2-Minute Walk Test.

Note. *: $p < 0.05$.

Normality was assessed using both the Kolmogorov-Smirnov test and visual inspection of histograms and Q-Q plots. Variables indicated with † were classified as approximately normal despite a significant Kolmogorov-Smirnov test because graphical assessment supported an approximately normal distribution.

Table S2: Results of the Kolmogorov-Smirnov normality test of standardized residuals.

**Regression model**	**Kolmogorov-Smirnov statistic (D)**	**p-value**
**Pain intensity**		
Model 1: Descriptive characteristics of subjects	0.128	0.063
Model 2: PPT	0.088	0.200
Model 3: Health status	0.109	0.200
**PPT**		
Model 1: Descriptive characteristics of subjects	0.143	0.031*
Model 2: Pain intensity	0.097	0.200
Model 3: Health status	0.072	0.200

Abbreviations. PPT: pressure pain threshold.

Note. *: $p < 0.05$.

Normality of standardized residuals was assessed using the Kolmogorov-Smirnov test complemented by visual inspection of histograms and Q-Q plots. Although one model showed a statistically significant Kolmogorov-Smirnov test ($p < 0.05$), visual inspection did not indicate meaningful deviations from normality. Given the sample size and the known sensitivity of the Kolmogorov-Smirnov test to minor deviations, residuals were considered approximately normally distributed and the assumptions of linear regression were deemed acceptable.
